# Proximate Composition, Antioxidant Capacity, and Dietary Fiber Functional Properties of Pulp and Peel of Two *Oenocarpus bataua* Morphotypes from the Bolivian Amazon: Effects of Preservation Methods and Frozen Storage

**DOI:** 10.3390/foods15162819

**Published:** 2026-08-13

**Authors:** Rosa F. Zabalaga, Daniela Sejas Pérez, Brenda Fortunata Villena Vargas, Marcos Luján Pérez, Freddy S. Zenteno Ruiz, Daniel M. Larrea-Alcázar

**Affiliations:** 1 Centro de Investigación en Ciencias Exactas e Ingeniería (CICEI), Departamento de Ciencias Exactas e Ingeniería, Universidad Católica Boliviana “San Pablo”, Cochabamba, Bolivia; danielasejas076@gmail.com (D.S.P.); brendavillena17@gmail.com (B.F.V.V.); mlujan@ucb.edu.bo (M.L.P.); 2 Herbario Nacional de Bolivia, Instituto de Ecología, Universidad Mayor de San Andrés (UMSA), La Paz, Bolivia; fred6zenruiz@gmail.com; 3 Programa de Ciencia y Tecnología, Asociación Boliviana para la Investigación y Conservación de Ecosistemas Andino-Amazónicos (Conservación Amazónica-ACEAA), La Paz, Bolivia; dlarrea@conservacionamazonica.org.bo

**Keywords:** bioeconomy, hot-air drying, freeze-drying, functional properties, postharvest storage, Amazonian palm, dietary fiber, storage, lipid content in pulp and peel, proximate composition

## Abstract

*Oenocarpus bataua* Mart. (Arecaceae) (known as “majo” in Bolivia) is an Amazonian palm fruit with considerable potential for the development of functional foods; however, its rapid postharvest deterioration limits its utilization. This study evaluated the effects of preservation methods and frozen storage on the physicochemical properties of two morphotypes of its fruits (dark-purple and pale-colored). Proximate analysis of pulp and peel fractions revealed a lipid-rich composition, with crude lipid contents close to 49% (dry basis), moderate protein levels (7.1–9.7%), and crude fiber contents around 15%. Dietary fiber characterization showed exceptionally high total dietary fiber (61.6%), predominantly insoluble dietary fiber (56.3%) under short-term frozen storage. Fresh pulp exhibited high antioxidant capacity, as determined by the DPPH assay (2003–2070 µmol TE/g dry matter), while freeze-drying preserved significantly higher antioxidant activity than hot-air drying. Functional properties were strongly affected by storage duration, with water-holding capacity decreasing from (4.43–3.17) g H_2_O/g dry matter and swelling capacity from 13.95 to 9.62 mL H_2_O/g dry matter after prolonged freezing. FTIR spectroscopy confirmed the presence of characteristic polysaccharide and lignocellulosic structures and indicated structural disruption of the insoluble fiber matrix during long-term storage. These findings support the use of Bolivian majo as a promising source of functional ingredients and provide a basis for developing value-added Amazonian food products and optimizing preservation strategies.

## 1. Introduction

**Figure 1:**
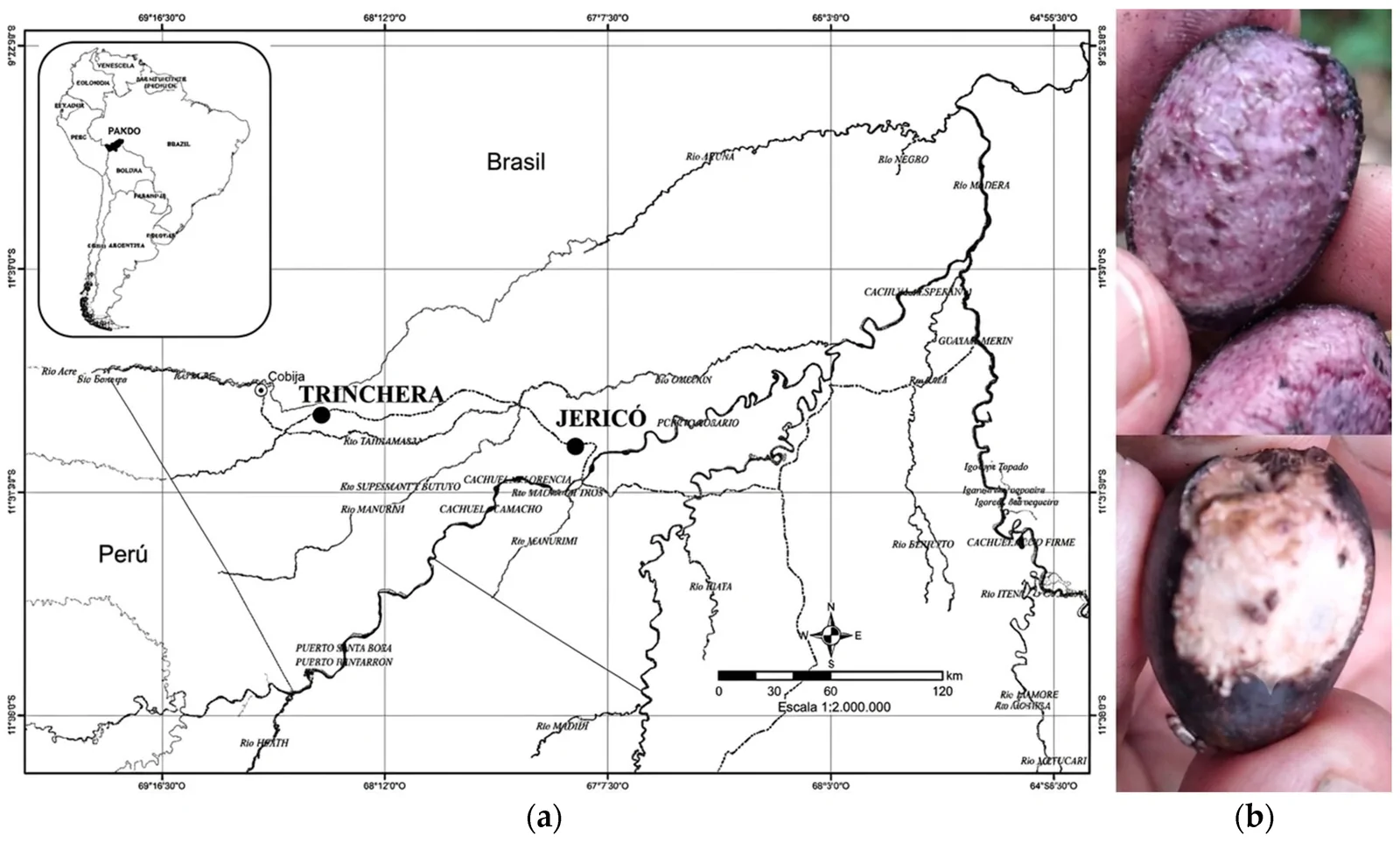
(**a**) Map of northern Bolivia showing the two rural communities of Jericó and Trinchera in Pando; (**b**) two morphotypes of fruits of *Oenocarpus batauaBataua* Mart. (Arecaceae): dark-purple and pale-colored morph.

In the northern Bolivian Amazon, particularly in the rural communities of Trinchera and Jericó (Figure 1a), *Oenocarpus bataua* Mart. (Arecaceae) (locally known as “majo”) is an important native resource with significant potential for the development of value-added products [1,2]. However, its utilization remains limited by structural and technological constraints. A major challenge lies in the high perishability of the fruit, which begins to deteriorate within 7–8 days post-harvest [3]. In regions with limited access to stable energy supply and processing infrastructure, the implementation of effective preservation strategies is essential to maintain both physicochemical properties and bioactive compounds. While hot-air dehydration represents a low-cost and accessible alternative, it may compromise heat-sensitive antioxidants compared to more advanced techniques such as freeze-drying.

Amazonian fruits are highly perishable due to their high moisture content and metabolic activity, limiting their shelf life and commercialization. Preservation technologies are therefore essential to reduce postharvest losses while retaining nutritional quality and bioactive compounds. Freezing is commonly applied for short- and medium-term storage, whereas freeze-drying provides superior preservation of heat-sensitive phytochemicals and hot-air drying offers a lower-cost alternative despite greater thermal degradation. Comparing these preservation methods is important for identifying suitable strategies to enhance the utilization and valorization of underutilized Amazonian fruits such as *Oenocarpus bataua*.

The adoption of advanced preservation technologies, such as lyophilization, could be supported by leveraging the existing infrastructure developed for the açaí industry, which has established processing facilities and commercial networks in the region. Integrating “majo” into these value chains would enable the preservation of its native polyphenolic profile, thereby enhancing its bioaccessibility and potential applications in nutraceutical and pharmaceutical sectors. Recent phytochemical investigations have positioned majo as a nutritionally and phytochemically valuable fruit due to its robust profile of bioactive compounds, including anthocyanins, condensed tannins, stilbenes, and phenolic acids. Its antioxidant capacity has been shown to match or even surpass that of açaí (*Euterpe oleracea* Mart.) in specific chemical assays, offering promising applications in the prevention of neurodegenerative and chronic inflammatory diseases [1,4].

Phenolic compounds have been reported as important contributors to the antioxidant activity of *O. bataua* and other Amazonian fruits. However, the present study evaluated antioxidant capacity rather than the concentration of individual bioactive compounds.

Dietary fiber has been associated with beneficial physiological effects such as improved intestinal function and regulation of blood glucose and cholesterol levels [5]. In this context, the dietary fiber of majo is of particular interest due to its potential functional properties. In northern Bolivia, local communities traditionally prepare a beverage known as “majo milk”, which is commonly consumed to alleviate digestive discomfort and sensations of gastric heaviness, suggesting a possible relationship between its fiber composition and gastrointestinal benefits.

*Oenocarpus bataua* populations exhibit visible phenotypic variation in fruit coloration, even within the same geographical zones [5]. In the northern Bolivian Amazon, specifically, in Pando, two morphotypes can be identified: a conventional dark-purple morph and a lighter, pale-colored morph (Figure 1b). These morphotypes coexist within the same regions and correspond to the same species, differing only in their external appearance. Such variation may be associated with differences in their biochemical composition, which are also evaluated in the present study. A comprehensive characterization of these morphotypes is therefore required to support their sustainable utilization under local conditions. This need is particularly relevant given the limited access to energy and processing infrastructure in rural communities, which constrains post-harvest handling and valorization strategies.

Accordingly, the objective of this study was to characterize the physicochemical properties, antioxidant capacity and dietary fiber profile of “majo” pulp obtained from the two coexisting morphotypes collected from two local communities (Trinchera and Jericó) in Pando, Bolivia. Additionally, the effect of selected preservation methods, including hot-air dehydration and freeze-drying, was evaluated to assess their suitability under both low-technology and more advanced processing scenarios. This approach provides scientific evidence to support the efficient utilization and valorization of both morphotypes of majo fruits as functional resources adapted to the technological constraints of the Bolivian Amazon, contributing to the development of high-value bio-based products.

## 2. Materials and Methods

### 2.1. Sample Collection

Majo fruits were sampled from two distinct rural communities within the department of Pando, Northern Bolivia: Trinchera (Nicolás Suárez Province) and Jericó (Manuripi Province) (Figure 1a). At both sites, two specific morphotypes—a pale-colored and a dark-purple variant—were collected. The exact geographic coordinates for the Trinchera specimens were 11°10′16.7″ S, 68°29′19.2″ W (pale) and 11°10′31.4″ S, 68°28′00.4″ W (dark-purple), while the Jericó samples were gathered at 11°17′46.1″ S, 67°16′08.1″ W (pale) and 11°19′53.4″ S, 67°15′15.75″ W (dark-purple). To preserve sample integrity, the harvested fruits were immediately placed in labeled black polyethylene bags and transferred to insulated polystyrene containers to mitigate light exposure and thermal degradation. Samples were subsequently frozen at −20 °C to preserve their physicochemical integrity and prevent degradation of bioactive compounds. The frozen samples were then transported by air from Pando to the laboratories in Cochabamba (central Bolivia) under controlled frozen conditions for further analysis. The frozen fruits were thawed, washed, and sanitized with 0.0055% sodium hypochlorite solution for 30 min. The fruits were then manually depulped, and the pulp and peel fractions were analyzed together. The samples were identified using the following codes: DPJ (dark-purple morph from Jericó), PCJ (pale-colored morph from Jericó), DPT (dark-purple morph from Trinchera), and PCT (pale-colored morph from Trinchera).

Fruits were collected during the local harvest season (April 2025), corresponding to the natural fruiting period of *Oenocarpus bataua* in the Bolivian Amazon. Fruits were harvested by experienced local collectors at the stage traditionally recognized as suitable for consumption and for the preparation of the regional beverage known as “majo milk,” corresponding to commercial maturity. Instrumental maturity indices (e.g., soluble solids, firmness, or peel color) were not determined because fruits were transported and stored under frozen conditions prior to analysis. Freezing and subsequent thawing may alter these parameters through tissue disruption, moisture redistribution, and solute leakage, making post-thaw measurements unsuitable for accurately assessing fruit maturity at harvest.

The pulp obtained after thawing the collected frozen fruits and before applying additional preservation treatments was considered the untreated frozen–thawed control (FT-control).

### 2.2. Proximate Composition

All analytical determinations were performed in triplicate on homogenized samples, and mean values were used for data comparison. Moisture, ash, crude lipid, and crude protein contents were determined according to AOAC methods 925.09, 942.05, 920.39, and 984.13, respectively. Crude protein was quantified by the Kjeldahl method using a nitrogen conversion factor of 6.25. Total carbohydrates were calculated by difference. Crude fiber content was determined according to AOAC method 978.10 using the Weende procedure. Approximately 1 g of previously dried and defatted sample was subjected to sequential acid digestion with 1.25% sulfuric acid (98%, Merck, Darmstadt, Germany) followed by alkaline digestion with 1.25% sodium hydroxide. The resulting residue was washed to neutral pH, dried at 105 °C for 2 h, and finally ashed at 500 °C for 3 h.

### 2.3. Soluble and Insoluble Dietary Fiber

Insoluble dietary fiber (IDF) and soluble dietary fiber (SDF) fractions were determined using an enzymatic–gravimetric procedure based on AOAC Official Method 2011.25, with modifications to the enzymatic digestion protocol. Briefly, approximately 1 g of sample was subjected to sequential enzymatic digestion to remove digestible components. Pancreatic α-amylase (Cat. No. A3176, Sigma-Aldrich, St. Louis, MO, USA) was first used to hydrolyze starch, followed by pepsin from porcine gastric mucosa (Cat. No. P7000, Sigma-Aldrich, St. Louis, MO, USA) for protein digestion. After enzymatic treatment, the hydrolysate was vacuum-filtered. The retained residue was washed with aqueous ethanol and acetone and recovered as the IDF fraction. The filtrate was mixed with ethanol to precipitate the SDF, which was subsequently recovered by centrifugation. Both fiber fractions were dried, quantified gravimetrically, and used to calculate total dietary fiber (TDF) as the sum of the IDF and SDF contents.

### 2.4. Water-Holding and Swelling Capacity

Water Holding Capacity (WHC) was determined following an adapted procedure from [6] widely used for the evaluation of dietary fiber functional properties. Briefly, a 0.08 M phosphate buffer solution at pH 6.1 was prepared. Subsequently, 0.1 g of IDF and SDF were weighed separately into centrifuge tubes, and 5 mL of phosphate buffer solution was added to each tube. The mixtures were manually stirred for approximately 10 min to ensure complete fiber hydration and then allowed to stand for 24 h at room temperature. Finally, the samples were centrifuged at 3000 rpm for 10 min, the supernatant was discarded, and the hydrated sediment was weighed.

Swelling capacity (SC) was determined according to [7]. Briefly, 0.1 g of dried IDF was weighed and transferred into a 10 mL graduated cylinder, and the initial dry volume occupied by the fiber was recorded. Subsequently, 5 mL of 0.08 M phosphate buffer solution (pH 6.1) was added to the cylinder. The mixture was manually stirred for approximately 10 min and allowed to stand at room temperature for 24 h to reach swelling equilibrium. Finally, the final volume occupied by the swollen sediment was measured.

### 2.5. FTIR Spectroscopy and Chemical Antioxidant Assays

FTIR spectroscopy was performed using a Shimadzu IR Prestige-21 spectrometer (Shimadzu, Kyoto, Japan, resolution 0.5 cm^−1^) to evaluate structural changes; cellulose pellets were prepared with KBr at a ratio of 1:100; 20 runs were recorded for each IR spectrum.

Antioxidant capacity was evaluated in pulp samples collected from both study locations and for both morphotypes (DPJ, PCJ, DPT and PCT). Additionally, antioxidant analyses were performed on samples preserved by freeze-drying and hot-air drying (45 and 55 °C) to assess the effect of the preservation method on bioactive compound retention and to determine whether the higher processing costs associated with freeze-drying are justified in terms of antioxidant preservation.

Antioxidant compounds were extracted according to the method described by [8]. Briefly, 0.5 g of sample was extracted with 20 mL of an acetone/ water mixture (1:1, *v*/*v*; 10 mL acetone and 10 mL water) under agitation for 3 h, followed by an additional extraction period of 12 h in darkness. The extracts were centrifuged at 3000 rpm for 10 min and filtered to recover the supernatant.

Antioxidant capacity was determined using the DPPH (2,2-diphenyl-1-picrylhydrazyl, Sigma-Aldrich, St. Louis, MO, USA) radical scavenging assay described by [9]. A DPPH solution was prepared in ethanol and adjusted to an absorbance of 0.8 at 515 nm. The reaction mixture consisted of 500 µL of extract, 300 µL of DPPH solution, and 3 mL of ethanol. After incubation for 30 min in darkness, absorbance was measured at 515 nm. Trolox (Sigma-Aldrich, St. Louis, MO, USA) was used as the reference standard, and a calibration curve was prepared using serial dilutions ranging from 0 to 200 µmol/L.

Antioxidant capacity was quantified from the Trolox calibration curve and expressed as micromoles of Trolox equivalents per gram of dry matter (µmol TE/g dm). The antioxidant concentration obtained for each extract was corrected for the total extraction volume and normalized to the dry weight of the analyzed sample. For the untreated frozen–thawed control pulp, antioxidant capacity values were converted to a dry matter basis using the experimentally determined moisture content obtained from proximate analysis, allowing direct comparison with freeze-dried and hot-air-dried samples.

### 2.6. Preservation Methods

The obtained pulp was freeze-dried using a BK-FD10P laboratory freeze dryer (BIOBASE, Jinan, Shandong, China) under vacuum conditions (≤5 Pa) with a cold-trap temperature of ≤−60 °C for 24–36 h until complete dehydration was achieved. Pulp thickness was measured in triplicate for each morphotype (dark-purple and pale-colored), with an average thickness of 2.77 ± 0.42 mm. Hot-air drying was carried out at 45 °C and 55 °C using a laboratory-scale hot-air tray dryer (Model ST-22, Shawtuuy, Shenzhen, China) operated at an air velocity of 2.5–3.0 m/s. The pulp was distributed in a single layer, and weight loss was monitored at 30 min intervals until constant weight was achieved, requiring approximately 700 min at 45 °C and 550 min at 55 °C. Drying completion was established gravimetrically based on constant sample weight; therefore, final moisture content was not measured as a process endpoint. Finally, the dried samples were ground using a laboratory mill, and both freeze-dried and hot-air-dried samples were used for all subsequent analyses.

### 2.7. Statistical Analysis

The obtained results were analyzed using Minitab software version 18.10.0 (Minitab Inc., State College, PA, USA). Analysis of variance (ANOVA) was performed to determine significant differences among treatments, followed by Tukey’s post hoc test for multiple comparisons and statistical grouping at *p* < 0.05. Antioxidant capacity data were analyzed using a three-way factorial ANOVA considering morphotype, collection region, and preservation method as fixed factors. When significant effects were detected, Tukey’s post hoc test was applied for pairwise comparisons. In addition, partial eta squared (partial η^2^) was calculated to estimate the effect size of each main factor and their interactions. Statistical significance was established at *p* < 0.05.

It is important to note that analyses of dietary fiber composition, including insoluble dietary fiber (IDF), soluble dietary fiber (SDF), functional properties [water-holding capacity (WHC) and swelling capacity (SC)], and FTIR characterization, were performed exclusively on the dark-purple morphotype collected in Jericó. This morphotype was selected as the reference material to evaluate the effects of frozen storage at −20 °C, comparing short-term frozen storage (STFS; analyses performed within 10 days of storage) with long-term frozen storage (LTFS; analyses performed after more than 6 months of storage), on dietary fiber composition, functional properties, and structural characteristics.

## 3. Results and Discussion

### 3.1. Proximate Characteristics

The results show a remarkably high lipid content, representing nearly half of the total composition on a dry basis (Table 1). Similar findings were reported by [3], who found a crude lipid content of approximately 47.0% (dry basis) in the pulp of *O. bataua* fruits collected in Peru. This similarity suggests that the high oil content is an intrinsic characteristic of the species, reinforcing its potential as a valuable source of edible oil and functional food ingredients.

The high lipid content observed in the present study is consistent with reports for other Amazonian palm fruits. In particular, ref. [10] reported a lipid content of approximately 30% (dry basis) in the peel of bacaba (*O. istichus*), another species within the *Oenocarpus* genus. Although direct comparisons should be interpreted with caution because of interspecific differences, these findings further support the oleaginous nature of *Oenocarpus* fruits and highlight their potential as valuable sources of edible oils. In the present study, the pulp and peel were analyzed together in order to evaluate the overall nutritional potential of the fruit and to promote its possible agro-industrial valorization. This approach is particularly relevant for northern Bolivia (Jericó and Trinchera), where processing facilities currently dedicated to harvesting of Brazil nut (*Bertholletia excelsa*) and wild açaí berry (*Euterpe precatoria*) could potentially incorporate majo processing, thereby creating new economic opportunities for local communities.

In contrast, crude fiber values obtained in the present work (≈15% db) may initially appear moderate when compared with the lipid fraction [1,2,11]. However, crude fiber determined by the Weende method is known to underestimate the actual dietary fiber content due to partial degradation of hemicellulose, lignin, and soluble polysaccharides [12]. For this reason, additional analyses of insoluble and soluble dietary fiber fractions were performed using the enzymatic–gravimetric AOAC 2011.25 method. On the other hand, protein contents in the Bolivian samples ranged from 7.13 to 9.73% dry basis, indicating a moderate protein contribution compared with the dominant lipid fraction. Meanwhile, carbohydrate contents remained relatively low, which is characteristic of lipid-rich Amazonian fruits where reserve compounds are preferentially accumulated as oils rather than starches or soluble sugars.

Overall, the proximal composition of majo fruits differed according to both morphotype and collection site. Among the evaluated samples, the dark-purple morph collected from Jericó (DPJ) exhibited the highest crude protein and crude fiber contents, while maintaining a lipid content comparable to the other dark-purple morph (DPT) and the pale-colored morph from Trinchera (PCT). In contrast, the pale-colored morph from Jericó (PCJ) showed the highest carbohydrate content and the lowest lipid concentration. These findings suggest that both fruit morphotype and geographical origin contribute to the nutritional variability of majo fruits, with the dark-purple morph from Jericó representing the most promising material for food and agro-industrial applications.

### 3.2. Antioxidant Capacity

As presented in Table 2, although freeze-drying resulted in a significant decrease (~35%) in antioxidant capacity compared to FT-control, it outperformed hot-air drying, which suffered even greater thermal degradation. In contrast, hot-air drying caused a progressive reduction in antioxidant activity, particularly at 55 °C. These findings suggest that thermal processing promotes the degradation of antioxidant compounds, whereas freeze-drying minimizes oxidative and thermal damage. Regardless of the preservation method, the dark-purple morphotype consistently exhibited higher antioxidant capacity than the pale-colored morphotype. This trend is likely associated with the greater accumulation of phenolic compounds and pigmented phytochemicals in dark-colored fruits, particularly anthocyanins and other flavonoids.

From a technological perspective, these findings highlight the trade-off between antioxidant retention and processing practicality. Freeze-drying provided the highest preservation of antioxidant capacity among the evaluated preservation methods, likely due to the absence of thermal degradation, whereas hot-air drying, despite being a simpler and more accessible preservation technique, resulted in greater losses of antioxidant activity as drying temperature increased. Consequently, the selection of an appropriate preservation strategy for *O. bataua* pulp should consider not only the retention of bioactive compounds but also the intended application of the final product and the technical and economic feasibility of the preservation process.

A consistent geographical effect was also observed, as samples collected in Jericó exhibited higher antioxidant activity than their counterparts from Trinchera. Environmental factors such as soil composition, solar radiation, water availability, and microclimatic conditions may influence the biosynthesis and accumulation of antioxidant metabolites in majo fruits.

**Table 2:** Effect of preservation method on the DPPH antioxidant capacity of *Oenocarpus bataua* (Arecaceae) pulp from two morphotypes collected in the Jericó and Trinchera regions of northern Bolivia.

Preservation Method	Antioxidant Capacity (DPPH) [µmol TE */g dm]			
	*Pataua* Pulp			
	DPJ	DPT	PCJ	PCT
FT-control	2070.05 ± 9.24 ^a^	2065.42 ± 42.23 ^a^	2004.68 ± 32.7 ^a^	2003.53 ± 42.45 ^a^
Freeze-dried	1362.96 ± 9.91 ^b^	1384.73 ± 8.25 ^b^	1321.27 ± 19.78 ^b^	1258.59 ± 24.95 ^b^
Hot-air-dried 45 °C	1211.44 ± 13.44 ^c^	1101.65 ± 28.58 ^c^	1072.98 ± 15.16 ^c^	1138.05 ± 5.01 ^c^
Hot-air-dried 55 °C	1122.35 ± 11.76 ^d^	990.33 ± 11.77 ^d^	1006.07 ± 18.40 ^c^	653.65 ± 13.35 ^d^

Mean ± SD; (*n* = 3). Different lowercase letters within each column indicate significant differences among preservation methods according to Tukey’s HSD test (*p* < 0.05). * Trolox equivalent.

The antioxidant capacities obtained for Bolivian majo pulp were comparable to those reported by [2] for fruits collected at different altitudes in Peru, ranging from approximately 1400 to 2200 µmol TE/g. This close agreement supports the reliability of the antioxidant values obtained in the present study. Similarly, ref. [4] reported antioxidant capacities of approximately 2300 µmol TE/g for majo fruits collected in French Guiana, values that are also consistent with those observed in the Bolivian samples.

In support of these findings, [13] reported antioxidant capacities of approximately 4900 [µmol TE/g] for açaí seeds and 1500 [µmol TE/g] for majo seeds from the northern Bolivian regions. The comparatively higher values observed in majo pulp suggest that the edible mesocarp may represent a more important reservoir of bioactive constituents than the seed fraction. This observation further highlights the nutritional and functional potential of majo pulp as a rich source of natural antioxidants and supports its utilization in the development of functional foods and nutraceutical products.

Nevertheless, comparisons among studies should be interpreted with caution, as antioxidant capacity is strongly influenced by extraction procedures, analytical methodologies, expression units, and sample preparation protocols. Despite these limitations, the consistently high antioxidant values observed in the present work reinforce the potential of Bolivian majo as a valuable source of bioactive compounds with promising applications in functional foods and nutraceutical products. The high antioxidant capacity may be associated with bioactive constituents previously reported for *O. bataua*, including phenolic compounds, although these were not quantified in the present study.

To further evaluate the factors influencing antioxidant capacity, a three-way factorial ANOVA was performed considering morphotype, collection region, and preservation method as fixed factors (Table 3). The analysis revealed that all three main factors significantly affected antioxidant capacity (*p* < 0.001). Among the evaluated factors, preservation method showed the strongest statistical effect on antioxidant capacity (F = 5709.816, *p* < 0.001), indicating that the preservation strategy was the primary determinant of antioxidant retention. Morphotype (F = 266.81, *p* < 0.001) and collection region (F = 141.20, *p* < 0.001) also had significant effects, demonstrating that both the intrinsic characteristics of the fruit and its geographical origin contributed to the observed variability. Overall, these findings indicate that antioxidant capacity is influenced by both preservation strategy and biological variability rather than by preservation method alone.

The interaction analysis provided additional biological insight. Significant Morphotype × Preservation Method (F = 43.62, *p* < 0.001), Collection Region × Preservation Method (F = 78.14, *p* < 0.001), and Morphotype × Collection Region × Preservation Method (F = 46.74, *p* < 0.001) interactions indicate that the response of antioxidant capacity to preservation differed according to both fruit morphotype and collection region. Thus, the magnitude of antioxidant retention or degradation depended on the specific combination of preservation method, morphotype, and geographical origin. In contrast, the Morphotype × Collection Region interaction alone was not significant (F = 3.93, *p* = 0.056), indicating that these two factors did not independently explain the observed variation in antioxidant capacity. Collectively, these results demonstrate that preservation strategies should be optimized according to both the biological characteristics of the fruit and its geographical origin rather than generalized across all *Oenocarpus bataua* populations.

Partial eta-squared (ηp^2^) values further supported the ANOVA results by quantifying the magnitude of each effect. Preservation method exhibited the largest effect size (ηp^2^ = 0.998), confirming that it was the primary determinant of antioxidant capacity. Morphotype (ηp^2^ = 0.893) and collection region (ηp^2^ = 0.815) also showed very large effect sizes, indicating substantial biological contributions to the observed variability. Likewise, the significant interaction terms presented large effect sizes (ηp^2^ = 0.804–0.880), demonstrating that the response to preservation depended strongly on the combined influence of fruit morphotype and geographical origin. In contrast, the Morphotype × Collection Region interaction showed a comparatively small effect (ηp^2^ = 0.109), consistent with its lack of statistical significance.

Although the present study focused on DPPH antioxidant capacity as a comparative indicator of preservation effects, future investigations should include complementary antioxidant assays (e.g., ABTS, FRAP and ORAC), together with total phenolic content, anthocyanin quantification and chromatographic profiling, to provide a more comprehensive characterization of the bioactive compounds present in *O. bataua* pulp.

### 3.3. Soluble, Insoluble Dietary Fiber, Water Holding and Water Swelling Capacity

Table 4 presents the contents of IDF, SDF and TDF in majo pulp samples collected corresponding to the dark-purple morphotype subjected to short-term (STFS) and long-term frozen storage (FTFS), respectively.

Majo pulp is an exceptionally rich source of dietary fiber, particularly insoluble dietary fiber (IDF). Although elevated dietary fiber levels could be expected in palm-derived fruits, the fiber contents observed in majo pulp are unusually high and are more commonly reported in food processing by-products rather than in the edible pulp itself. These findings highlight the nutritional and functional potential of majo pulp as a promising Amazonian raw material for the development of novel functional and value-added food products [14,15,16].

Total dietary fiber (TDF) reached 61.60% under short-term frozen storage and remained relatively high (35.80%) even after prolonged storage at −20 °C. These values are considerably higher than those reported for many conventional plant matrices such as apple, banana, and oats [17]. The predominance of IDF indicates that majo pulp is a highly structural lignocellulosic matrix, insoluble polysaccharides (cellulose and hemicellulose) and soluble polysaccharides (pectin and β-glucans) [17,18], which is biologically consistent with its nature as an oleaginous palm fruit with thick cell walls. From a nutritional perspective, the high IDF content may contribute to improved intestinal transit, increased fecal bulk, water retention, laxative effects, and enhanced satiety. These findings may also support the traditional use of “majo milk” by local communities in northern Bolivia as a beverage associated with digestive relief and reduced gastric heaviness.

A pronounced reduction in IDF content was observed after prolonged frozen storage, decreasing from 56.28% to 30.90%, corresponding to an approximate 45% reduction. In contrast, SDF values remained comparatively stable, resulting in an increased SDF/IDF ratio after long-term storage. This behavior is fundamentally driven by the physical and enzymatic degradation of the plant cell wall matrix. Prolonged storage at −20 °C promotes the continuous growth of large extracellular and intracellular ice crystals, generating severe hydromechanical stress that physically disrupts cellulose microfibrils and collapses the structural network [19]. Furthermore, this cellular collapse destroys vacuolar compartmentalization, releasing latent endogenous hydrolytic enzymes—such as cellulases and pectinases—that progressively hydrolyze insoluble structural polymers over time [20]. This disruption weakens the hydrogen bonds and calcium bridges anchoring the hemicelluloses within the insoluble matrix, leading to partial depolymerization and subsequent leaching or migration of these structural fractions into more soluble or matrix-free exudates during storage [21,22,23]. Despite representing a smaller fraction than insoluble dietary fiber, soluble dietary fiber still plays an important role in regulating nutrient absorption and helping reduce blood cholesterol and glucose levels [24,25,26].

Statistical analysis confirmed significant differences (*p* < 0.05) between short-term and long-term frozen storage for IDF, TDF, WHC, and SC. In contrast, SDF content did not show statistically significant differences between storage conditions (*p* > 0.05). These results indicate that prolonged frozen storage mainly affected the insoluble dietary fiber fraction and its associated functional properties.

It should be noted that dietary fiber composition, functional properties, and FTIR characterization were evaluated exclusively in the dark-purple morphotype collected from Jericó. Consequently, although these findings provide valuable insight into the effects of frozen storage on the structural and functional properties of majo dietary fiber, they should not be directly generalized to other morphotypes or collection sites without further investigation. Future studies including additional morphotypes and geographical origins are needed to determine the extent to which these responses are representative of *O. bataua* fruits from the Bolivian Amazon.

In this context, the assessment of WHC provides critical insights into the fiber’s capacity to modulate viscosity and physiological behavior throughout the gastrointestinal tract—factors directly involved in glycemic regulation, fermentability, and satiety mechanisms. Furthermore, an elevated WHC indicates a higher prevalence of soluble or less-ordered insoluble polysaccharides capable of forming extensive hydrated matrices, which carries significant nutritional and metabolic implications. For comparison, WHC values reported for citrus fiber range from around 1.6 to almost 13 [g H_2_O/g dm], whereas, for cereal by-products, it ranges from 3 to 3.8 [g H_2_O/g] [16,17,18].

The WHC values obtained for majo pulp ranged from 4.43 to 3.17 [g H_2_O/g dm], indicating a relatively high hydration capacity compared with several conventional fiber sources. These results suggest the presence of dietary fiber components capable of retaining substantial amounts of water and forming hydrated matrices, which may contribute to physiological effects associated with satiety, intestinal functionality, and modulation of nutrient absorption [17,20].

The SC of dietary fiber may increase gastrointestinal viscosity, which can delay nutrient absorption and help attenuate postprandial blood glucose and insulin levels [27,28]. The SC values obtained for “majo” pulp ranged from 9.62 to 13.95 [mL/g dm], indicating a remarkably high swelling behavior compared with several conventional dietary fiber sources, including mango, pineapple, and other tropical fruits [26,27,28,29]. These elevated SC values suggest that the insoluble dietary fiber fraction of majo possesses a strong hydration and expansion capacity, highlighting its potential as a valuable functional fiber source for food and nutritional applications.

### 3.4. FTIR Spectroscopy

**Figure 2:**
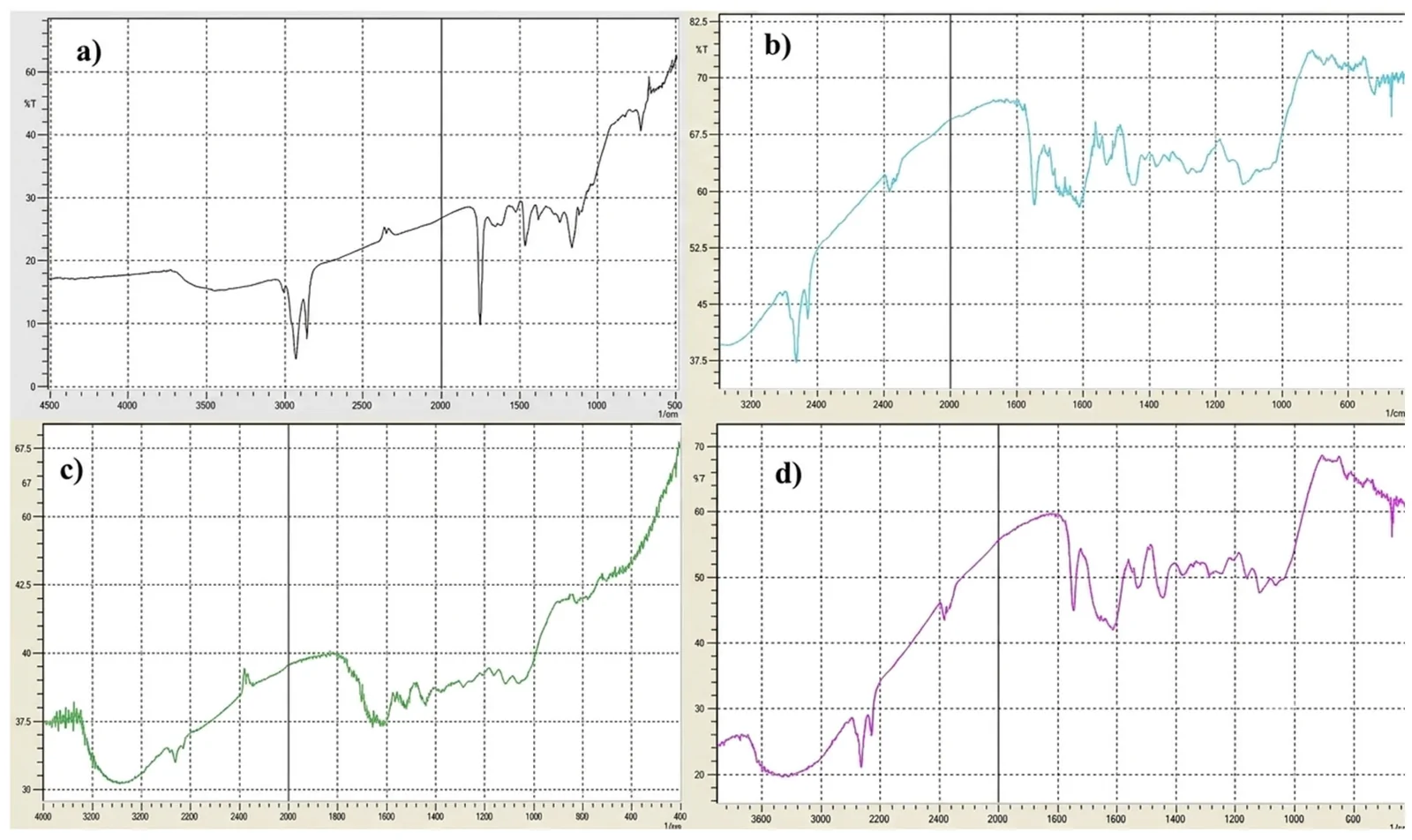
Fourier transform infrared (FTIR) spectra of *Oenocarpus bataua* (Arecaceae) pulp dietary fiber: on the left, IDF at short-term (**a**) and long term (**c**) frozen storage; on the right, SDF at short-term (**b**) and long term (**d**) frozen storage, respectively.

The FTIR spectra of insoluble dietary fiber (IDF) (Figure 2a,c) and soluble dietary fiber (SDF) (Figure 2b,d) obtained from majo pulp subjected to short-term and long-term frozen storage, respectively, revealed absorption bands consistent with those commonly reported for polysaccharides, lignocellulosic structures, and hydrogen-bonded functional groups commonly found in plant dietary fibers. Similar spectral regions have been reported for dietary fibers extracted from cassava pulp and other plant matrices [30,31,32].

Altogether, all spectra exhibited characteristic absorption regions commonly associated with plant dietary fibers, particularly within the regions of 2920–2850 cm^−1^ and 1200–900 cm^−1^. The bands near 2920–2850 cm^−1^ are attributed to the asymmetric and symmetric stretching vibrations of aliphatic C–H groups, characteristic of cellulose and hemicellulose associated polysaccharides, although residual lipid-derived aliphatic chains may also contribute in this oleaginous palm fruit. Similar assignments have been reported for fruit dietary fibers and plant cell wall polysaccharides [33]. Meanwhile, the fingerprint region between 1200 and 900 cm^−1^ showed signals associated with C–O, C–O–C, and glycosidic bond vibrations characteristic of structural polysaccharides such as cellulose and hemicellulose. This spectral region is widely recognized as one of the most informative for the characterization of plant cell wall polysaccharides, including cellulose, hemicelluloses, and pectic polysaccharides, and has been extensively used to characterize dietary fiber from fruit matrices by FTIR spectroscopy [33,34,35,36].

A broad absorption band between approximately 3600 and 3000 cm^−1^ was particularly evident in the IDF spectrum obtained after long-term frozen storage (Figure 2c). This region is commonly assigned to O–H stretching vibrations arising from hydroxyl groups of polysaccharides and hydrogen-bonded water molecules retained within the fiber matrix, as previously reported for plant cell wall polysaccharides and dietary fibers [34,36]. Although FTIR does not provide a direct quantitative measure of water-holding capacity, the presence of this broad band is consistent with the hydrophilic nature of dietary fiber and supports the experimentally determined water retention capacity (WHC) of the insoluble fraction.

Among all spectra, the IDF obtained after short-term frozen storage (Figure 2a) exhibited the most pronounced and complex spectral profile, particularly in the 1650–900 cm^−1^ region. The strong absorption band near 1030–1050 cm^−1^ suggests a highly preserved polysaccharide network rich in glycosidic structures and lignocellulosic components. This behavior agrees with the significantly higher IDF content, WHC, and SC experimentally observed under short-term frozen storage conditions.

In contrast, the spectrum corresponding to IDF after long-term frozen storage (Figure 2c) appeared less defined and exhibited lower spectral complexity, suggesting partial disruption of the structural fiber matrix. Nevertheless, the persistence of the major absorption bands indicates that the fundamental chemical composition of the fiber was preserved, suggesting that freezing mainly affected the physical organization of the fiber matrix, which may also have influenced the analytical recovery of the insoluble fraction, rather than causing extensive chemical degradation. Additional structural analyses would be required to confirm the underlying mechanisms.

The SDF spectra (Figure 2b,d) exhibited comparatively smaller variations between storage conditions, which is in agreement with the relatively stable SDF values experimentally obtained. This observation suggests that the soluble fiber fraction was less susceptible to structural changes induced by prolonged frozen storage than the insoluble fraction. Overall, the FTIR spectra were consistent with the physicochemical results, showing that the largest spectral differences occurred in the insoluble fiber fraction, which also exhibited the greatest reductions in IDF, WHC, and SC after prolonged frozen storage.

Although prolonged frozen storage resulted in a marked reduction in IDF content, no corresponding increase in SDF was observed. This finding suggests that the decrease in the insoluble fraction was not necessarily associated with its conversion into soluble dietary fiber. Instead, freezing- and thawing-induced disruption of the fiber matrix may have generated partially degraded or low-molecular-weight polysaccharide fragments that were not efficiently recovered by the enzymatic–gravimetric method. This interpretation is consistent with the FTIR results, which indicate modifications in the physical organization of the insoluble fiber matrix while preserving its main chemical functional groups. Nevertheless, further studies using complementary structural and molecular analyses are required to elucidate the mechanisms responsible for these changes.

It should be emphasized that FTIR spectroscopy provides information on the structural organization and functional groups present in the dietary fiber matrix rather than the direct identification or quantification of individual bioactive compounds. Therefore, the spectral assignments presented here should be interpreted as evidence consistent with structural polysaccharides and lignocellulosic components previously reported for plant dietary fibers.

## 4. Conclusions

The two morphotypes of *O. bataua* exhibited a remarkably high lipid content, representing nearly half of the fruit dry matter, confirming the oleaginous nature of majo and highlighting its potential as a valuable source of edible oil and energy-rich food ingredients. In contrast, protein and carbohydrate contents were comparatively lower, consistent with the compositional profile of Amazonian palm fruits. Freeze-drying proved to be the most effective preservation method for maintaining antioxidant capacity, yielding values statistically comparable to those of FT-control. The dark-purple morphotype consistently exhibited higher antioxidant activity than the pale-colored morphotype, while fruits collected in Jericó showed greater antioxidant potential than those from Trinchera, suggesting the influence of both phenotypic and environmental factors on bioactive compound accumulation. These findings provide useful information for selecting appropriate preservation strategies for processing and commercializing majo-derived products.

Majo pulp was identified as an exceptionally rich source of dietary fiber, particularly insoluble dietary fiber. Although prolonged frozen storage significantly reduced IDF, TDF, water retention capacity, and swelling capacity, the fiber content remained comparatively high relative to many conventional plant sources. These findings support the traditional use of majo-derived beverages for digestive well-being and highlight the functional potential of the fruit as a source of dietary fiber. FTIR analysis confirmed the predominance of structural polysaccharides associated with insoluble dietary fiber, including cellulose and hemicellulose related functional groups. The spectral profiles suggest that prolonged frozen storage may alter the organization of the fiber matrix without completely modifying its fundamental chemical structure. The absence of a concomitant increase in soluble dietary fiber despite the marked reduction in insoluble dietary fiber further suggests that prolonged frozen storage primarily affects the structural organization and analytical recovery of the fiber matrix rather than promoting a direct conversion of insoluble into soluble dietary fiber. However, complementary structural and molecular studies are required to confirm the mechanisms underlying these changes.

Overall, this work contributes new knowledge on the physicochemical and functional properties of Bolivian majo and supports its sustainable utilization as a source of high-value food ingredients. Future research should investigate the bioaccessibility of antioxidant compounds and dietary fiber, characterize the lipid fraction in greater detail, and evaluate the feasibility of scaling up preservation technologies for industrial applications.

## Figures and Tables

**Table 1:** Comparative proximate composition of *Oenocarpus bataua* (Arecaceae) pulp from dark-purple and pale-colored morphotypes collected in the Jericó and Trinchera regions of northern Bolivia.

Parameters	Majo Pulp + Peel			
	DPJ	DPT	PCJ	PCT
Moisture	39.28 ± 0.28 ^a^	37.01 ± 0.06 ^b^	36.77 ± 0.04 ^c^	37.29 ± 0.49 ^b^
Ash	3.23 ± 0.21 ^ab^	3.35 ± 0.08 ^a^	3.18 ± 0.01 ^b^	3.22 ± 0.03 ^ab^
Crude lipids	49.37 ± 0.23 ^a^	49.33 ± 0.08 ^a^	48.02 ± 0.07 ^b^	49.02 ± 0.39 ^a^
Crude protein	9.73 ± 0.07 ^a^	7.46 ± 0.14 ^b^	7.45 ± 0.21 ^b^	7.13 ± 0.1 ^b^
Crude fiber	15.69 ± 0.07 ^a^	15.02 ± 0.02 ^b^	14.77 ± 0.01 ^c^	14.81 ± 0.12 ^c^
Total carbohydrates	21.95 ^c^	24.86 ^b^	26.57 ^a^	25.75 ^ab^

Results are expressed on a dry weight basis, except for moisture content expressed on a fresh weight basis. Values are means ± SD (*n* = 3). Different superscript letters within the same row indicate statistically significant differences (*p* < 0.05) according to Tukey’s HSD test. DPJ: Dark-purple morph from Jericó; DPT: dark-purple morph from Trinchera; PCJ: pale-colored morph from Jericó; PCT: pale-colored morph from Trinchera.

**Table 3:** Three-way factorial ANOVA and effect size (partial η^2^) for antioxidant capacity of *Oenocarpus bataua* Mart. (Arecaceae) pulp as affected by morphotype, collection region, and preservation method.

Source of Variation	df	F	*p*-Value	Partial ηp^2^
Morphotype	1	266.808	<0.001	0.893
Collection region	1	141.2	<0.001	0.815
Preservation method	3	5709.816	<0.001	0.998
Morphotype × Collection region	1	3.933	<0.001	0.109
Morphotype × Preservation method	3	43.623	<0.001	0.804
Collection region × Preservation method	3	78.140	<0.001	0.88
Morphotype × Collection region × Preservation method	3	46.739	<0.001	0.814
Residual error	32	-	-	-
Total	47	-	-	-

df, degrees of freedom; F, F-statistic; *p*, probability value; partial η^2^, partial eta squared (effect size). Partial η^2^ values were used to estimate the magnitude of the effect of each factor and their interactions.

**Table 4:** Insoluble dietary fiber (IDF), soluble dietary fiber (SDF), and total dietary fiber (TDF) water holding capacity (WHC), and swelling capacity (SC) in *Oenocarpus bataua* Mart. (Arecaceae) dark-purple morphotype pulp subjected to short-term (STFS) and long-term frozen storage (LTFS) at −20 °C.

Storage Condition	IDF (%)	SDF (%)	TDF (%)	IDF-WHC (g H_2_O/g dm)	SDF-WHC (g H_2_O/g dm)	IDF-SC (mL_sed_/g dm) *
STFS	56.28 ± 0.85 ^a^	5.32 ± 0.82 ^a^	61.6 ± 0.88 ^a^	6.33 ± 0.28 ^a^	4.43 ± 0.28 ^a^	13.95 ± 1.17 ^a^
LTFS	30.90 ± 0.96 ^b^	4.90 ± 1.18 ^a^	35.80 ± 1.21 ^b^	4.47 ± 0.3 ^b^	3.17 ± 0.09 ^b^	9.62 ± 1.18 ^b^

Values are expressed as mean ± standard deviation of analytical triplicate measurements performed on homogenized samples. Different superscript letters within the same column indicate significant differences according to Tukey’s test (*p* < 0.05). * [mLsed/g dm] milliliters of swollen sediment per gram of dry matter.

## Data Availability

The data presented in this study are available from the corresponding author upon reasonable request.

## Abbreviations

The following abbreviations are used in this manuscript:

DPJ	dark-purple morph from Jericó
PCJ	pale-colored morph from Jericó
DPT	dark-purple morph from Trinchera
PCT	pale-colored morph from Trinchera
IDF	Insoluble Dietary Fiber
SDF	Soluble Dietary Fiber
TDF	Total Dietary Fiber
WHC	Water Holding Capacity
SC	Swelling Capacity
FTIR	Fourier Transform Infrared Spectroscopy
STFS	Short-Term Frozen Storage
LTFS	Long-Term Frozen Storage

## References

[1] Da Silva N.C.S., De Meneses Costa Ferreira L.M., Lynch D.G., Júnior J.O.C.S., Costa R.M.R. (2026). An In-Depth Review of the Phytochemical Profile and Pharmacological Potential of *Oenocarpus bataua*. Phytochem. Rev..

[2] Vargas-Arana G., Merino-Zegarra C., del-Castillo Á.M.R., Quispe C., Viveros-Valdez E., Simirgiotis M.J. (2022). Antioxidant, Antiproliferative and Anti-Enzymatic Capacities, Nutritional Analysis and UHPLC-PDA-MS Characterization of Ungurahui Palm Fruits (*Oenocarpus bataua* Mart) from the Peruvian Amazon. Antioxidants.

[3] Cotos M.R.C., Hameed I.H., Escajadillo S.B.E., Llica E.R., Figueroa M.G.R., Garcia J.E.O. (2020). Macronutrients, Polyphenols and Antioxidant Capacity of the Peel and Pulp of the Fruit *Oenocarpus bataua* Mart. “Ungurahui”. Res. J. Pharm. Technol..

[4] Rezaire A., Robinson J.-C., Bereau D., Verbaere A., Sommerer N., Khan M.K., Durand P., Prost E., Fils-Lycaon B. (2014). Amazonian Palm *Oenocarpus bataua* (“Patawa”): Chemical and Biological Antioxidant Activity—Phytochemical Composition. Food Chem..

[5] Peralta C., Miranda J., Moraes R.M., Moraes R.M. (2020). Oenocarpus bataua: Una palmera aprovechada a nivel regional. Palmeras y Usos: Especies de Bolivia y la Región.

[6] Robertson J.A., Eastwood M.A. (1981). A Method to Measure the Water-Holding Properties of Dietary Fibre Using Suction Pressure. Br. J. Nutr..

[7] Robertson J.A., De Monredon F.D., Dysseler P., Guillon F., Amado R., Thibault J.-F. (2000). Hydration Properties of Dietary Fibre and Resistant Starch: A European Collaborative Study. LWT—Food Sci. Technol..

[8] Xu B.J., Chang S.K.C. (2007). A Comparative Study on Phenolic Profiles and Antioxidant Activities of Legumes as Affected by Extraction Solvents. J. Food Sci..

[9] Brand-Williams W., Cuvelier M.E., Berset C. (1995). Use of a Free Radical Method to Evaluate Antioxidant Activity. LWT—Food Sci. Technol..

[10] Correa B.M., Baldissera L., Barbosa F.R., Ribeiro E.B., Andrighetti C.R., Agostini J.D.S., Valladão D.M.D.S. (2019). Centesimal and Mineral Composition and Antioxidant Activity of the Bacaba Fruit Peel. Biosci. J..

[11] Tauchen J., Bortl L., Huml L., Miksatkova P., Doskocil I., Marsik P., Villegas P.P.P., Flores Y.B., Damme P.V., Lojka B. (2016). Phenolic Composition, Antioxidant and Anti-Proliferative Activities of Edible and Medicinal Plants from the Peruvian Amazon. Rev. Bras. Farmacogn..

[12] Pesti G.M. (2024). A New Analytical Procedure to Replace the Outdated Weende Proximal Feed Ingredient Analysis Paradigm Is Long Overdue. Anim. Prod. Sci..

[13] Rosales C., Mejía C., Luján M., Zabalaga R., Larrea-Alcázar D.M. (2026). Physicochemical Characterization and Valorization Potential of Açaí Berry (*Euterpe precatoria*) and Bataua (*Oenocarpus bataua*) Seeds: Lignocellulosic Biomass and Antioxidants from Bolivian Amazon Agroindustrial Residues. Front. Nutr..

[14] de Almeida A.F. (2020). Frutos Amazônicos.

[15] Aguiar J.P.L., Souza F.D.C.D.A. (2014). Soluble and Insoluble Fiber in Some Amazonian Fruits with Low Energy Density. Food Nutr. Sci..

[16] Lima A.D.S., Cruz T.M., Silva A.O., Cox T., Dias J.V.B., Bezerra J.D.A., Lima M.D.S., Crispim M., Azevedo L., Magnani M. (2025). Amazonian Pataua (*Oenocarpus bataua*) Pulp Extract: Bioaccessibility of Phenolic Compounds and Effects on Cellular Antioxidant and Antimalarial Activities. Future Foods.

[17] Mammolenti D., Lupi F.R., Baldino N., Gabriele D. (2025). Technological Advancements of Insoluble Dietary Fiber from Food By-Product Processing: A Review. Foods.

[18] Qin W., Sun L., Miao M., Zhang G. (2021). Plant-Sourced Intrinsic Dietary Fiber: Physical Structure and Health Function. Trends Food Sci. Technol..

[19] Schudel S., Prawiranto K., Defraeye T. (2021). Comparison of Freezing and Convective Dehydrofreezing of Vegetables for Reducing Cell Damage. J. Food Eng..

[20] Wang Y., Cao X., Li Q., Nian R., You K., Su J., Xiong X., Zhu D. (2025). Effect of Freezing-Induced Pectin Degradation on the Texture Stability of Strawberry. Food Chem..

[21] Grover Y., Negi P.S. (2023). Recent Developments in Freezing of Fruits and Vegetables: Striving for Controlled Ice Nucleation and Crystallization with Enhanced Freezing Rates. J. Food Sci..

[22] Chassagne-Berces S., Fonseca F., Citeau M., Marin M. (2010). Freezing Protocol Effect on Quality Properties of Fruit Tissue According to the Fruit, the Variety and the Stage of Maturity. LWT—Food Sci. Technol..

[23] Elfalleh W., Guo L., He S., Wang P., Cui J., Ma Y. (2015). Characteristics of Cell Wall Structure of Green Beans During Controlled Freezing Point Storage. Int. J. Food Prop..

[24] Hernández Ramírez A., Gutiérrez Victorino K.G., Ariza Ortega J.A., Cruz Cansino N.D.S., Ariza Ortega T.E. (2026). Evaluación de Los Beneficios de La Fibra Dietética En Personas Con Enfermedades Cardiovasculares. Cienc. Lat. Rev. Científica Multidiscip..

[25] Guimarães A.D.A., Santos M.M., Jesus M.C.D., Haddad M.F., Ramos R.B., Freitas V.D.D. (2026). O Papel da Fibra na Regulação do Açúcar no Sangue e Sua Ação para Indivíduos Pré-Diabéticos. Rev. Ft.

[26] Tejada-Ortigoza V., Garcia-Amezquita L.E., Serna-Saldívar S.O., Welti-Chanes J. (2016). Advances in the Functional Characterization and Extraction Processes of Dietary Fiber. Food Eng. Rev..

[27] Kosmala M., Milala J., Karlińska E. (2025). Polysaccharide Composition of Dietary Fiber During Raspberry and Blackberry Juice Production. Molecules.

[28] Tan C., Wei H., Zhao X., Xu C., Peng J. (2017). Effects of Dietary Fibers with High Water-Binding Capacity and Swelling Capacity on Gastrointestinal Functions, Food Intake and Body Weight in Male Rats. Food Nutr. Res..

[29] Chen J., Gao D., Yang L., Gao Y. (2013). Effect of Microfluidization Process on the Functional Properties of Insoluble Dietary Fiber. Food Res. Int..

[30] Choct M. (2015). Feed Non-Starch Polysaccharides for Monogastric Animals: Classification and Function. Anim. Prod. Sci..

[31] Okrathok S., Thumanu K., Pukkung C., Molee W., Khempaka S. (2022). Extraction of Dietary Fibers from Cassava Pulp and Cassava Distiller’s Dried Grains and Assessment of Their Components Using Fourier Transform Infrared Spectroscopy to Determine Their Further Use as a Functional Feed in Animal Diets. Anim. Biosci..

[32] Dhingra D., Michael M., Rajput H., Patil R.T. (2012). Dietary Fibre in Foods: A Review. J. Food Sci. Technol..

[33] Hong T., Yin J.-Y., Nie S.-P., Xie M.-Y. (2021). Applications of Infrared Spectroscopy in Polysaccharide Structural Analysis: Progress, Challenge and Perspective. Food Chem. X.

[34] Chylińska M., Szymańska-Chargot M., Kruk B., Zdunek A. (2016). Study on Dietary Fibre by Fourier Transform-Infrared Spectroscopy and Chemometric Methods. Food Chem..

[35] Wan Y.-J., Hong T., Shi H.-F., Yin J.-Y., Koev T., Nie S.-P., Gilbert R.G., Xie M.-Y. (2021). Probiotic Fermentation Modifies the Structures of Pectic Polysaccharides from Carrot Pulp. Carbohydr. Polym..

[36] Resende L.M., Oliveira L.S., Franca A.S. (2020). Characterization of Jabuticaba (*Plinia cauliflora*) Peel Flours and Prediction of Compounds by FTIR Analysis. LWT.

